# Treating symptomatic uterine fibroids with myomectomy: current practice and views of UK consultants

**DOI:** 10.1186/s10397-017-1014-4

**Published:** 2017-07-06

**Authors:** R. Fusun Sirkeci, Anna Maria Belli, Isaac T. Manyonda

**Affiliations:** 1 St George’s, University of London, London, UK; 2 Department of Radiology, St George’s Healthcare NHS Foundation Trust, St George’s, University of London, London, UK; 3 Department of Obstetrics and Gynecology, St George’s Healthcare NHS Foundation Trust, St George’s, University of London, Blackshaw Road, Tooting, SW17 0QT London, UK

**Keywords:** Fibroids, Myomectomy, UAE, GnRHa, Vasopressin, Consultant practices

## Abstract

**Background:**

The demand for uterus-sparing treatments is increasing as more women postpone childbirth to their 30–40s, when fibroids are more symptomatic. With an increasing choice of treatment options and changing care-provider profiles, now is an opportune time to survey current practices and opinions. Using a 25-stem questionnaire, a web-based survey was used to capture the practices and opinions of UK consultant gynecologists on the treatment of symptomatic fibroids, including the types of procedure most frequently used, methods used to reduce blood loss, and awareness and acceptability of treatment options, and to assess the impact of gender and experience of the treating gynecologist.

**Results:**

The response rate was 22%. Laparascopic myomectomy is used least frequently, with 80% of the respondents using GnRHa preoperatively to minimize blood loss and correct anemia, while vasopressin is most frequently used to reduce intraoperative blood loss. Female consultants operate significantly less frequently than males. Those with more than 10 years consultant experience are more likely to perform an open myomectomy compared to those with less than 10 years experience.

**Conclusions:**

Compared to a similar survey performed 10 years ago, surgical methods remain to be the most common treatments, but use of less invasive treatments such as UAE has increased. Consultants’ attitudes appear to be responding to the patient demand for less radical treatments. However, it is yet to be seen if the changing consultant demographics will keep up with this demand. The low response rate warrants cautious interpretation of the results, but they provide an interesting snapshot of current views and practices.

**Electronic supplementary material:**

The online version of this article (doi:10.1186/s10397-017-1014-4) contains supplementary material, which is available to authorized users.

## Background

Fibroids are the most common tumor in women of reproductive age [1, 2]. Recent years have seen a demographic shift in childbirth trends, with many women delaying starting their families until they reach their third or fourth decade [3, 4]. This is the age when fibroids are more prevalent and symptomatic [5, 6]. The old adage “children then fibroids and then hysterectomy” therefore no longer applies to many a modern woman, and there is likely to be an increasing demand for fertility-preserving treatments for symptomatic uterine fibroids.

The repertoire of uterus-preserving treatments for symptomatic fibroids has increased in recent years. Just over 20 years ago, the use of uterine artery embolization (UAE) was first reported [7]. The National Institute for Health and Clinical Excellence (NICE) has reviewed its efficacy and recommends UAE as an alternative treatment to hysterectomy and myomectomy [8]. Magnetic resonance (MR)-guided focused ultrasound (MRgFUS) [9] is another new technique, but its adoption has been slow, partly due to the high infrastructure costs of setting up such a service, and because of its limitations in treating large and/or numerous fibroids [10, 11]. Pharmaceutical agents continue to be developed. While it was originally introduced as a pre-myomectomy treatment [12], ulipristal acetate has recently acquired a license for use as a stand-alone treatment for symptomatic fibroids [13] and is regarded by many as the “first-in-class” medical therapy for fibroids [14]. Despite the emergence of these new and exciting treatments for managing symptomatic uterine fibroids, in reality, when the uterus is to be preserved, myomectomy, especially the open abdominal approach, remains the treatment of choice of many gynecologists.

Surveys can be a useful tool in the evaluation of healthcare, whether by the recipients of healthcare or the healthcare professionals themselves. Historically, surveys were predominantly paper based, but the advent of the Internet and the development of dedicated software such as SurveyMonkey have made surveys much more efficient and powerful tools. With the new treatment modalities now embedding, and the changing demographics of both the profile of gynecological consultants and the population they serve, we considered it an opportune time to survey the views and practices of current UK consultant gynecologists on uterus-sparing techniques. We conducted an online survey and present and compare our findings to a similar paper-based survey conducted 10 years ago [15].

## Methods

Using a web-based survey system (SurveyMonkey, Inc.) a 25-item questionnaire was designed that included questions on the respondents’ gender, current place of work (i.e., district general hospital or tertiary center), how long they had been in a consultant role, and current clinical interests and subspecialty. The questionnaire further sought the respondents views and practices on the three types of myomectomy (open, laparoscopic, and hysteroscopic) and methods used to reduce blood loss both pre- and perioperatively. Additional questions were enquired about the respondents’ access to, and use of, cell salvage, and their views on UAE and ulipristal acetate (UPA). To refine and reduce ambiguity, the questionnaire was piloted among local gynecology consultants before invitations to participate were sent out nationally. A copy of the final version of the questionnaire is included in the Additional file 1.

The questionnaires were completed anonymously. The survey was active between November 2014 and April 2015. To maximize the response rate, the survey was advertised in the Royal College of Obstetricians and Gynecologists (RCOG) monthly electronic newsletter, the Scanner, which is e-mailed to all RCOG members. Two months after, the survey opened an invitation to participate and the link to the survey was also e-mailed to members of the British Society for Gynecological Endoscopy (BSGE).

As the study did not involve patients or their data, ethical approval was not required.

Statistical analysis was performed using SPSS software (IBM SPSS Statistics V22, Chicago, IL). The results are expressed in percentages (%) and absolute values (*n*). A *p* value equal to or less than 0.05 was considered significant.

## Results

### Response rate

Of the 1375 consultants invited to participate in the survey, 299 responded, a response rate of 22%.^1^ Among the respondents, a minority did not answer every question applicable to them. Therefore, for some questions discussed below, the total numbers are smaller than 299.

### Demographics

Thirty-six percent (*n* = 108) of respondents were female and 51% (*n* = 152) were male while 13% (*n* = 39) did not to disclose their gender. This correlates well with the actual gender distribution of current consultants in practice and gives some credence that the responses in our survey are representative of the workforce as a whole: as of 2013, 47% of the consultant workforce in the UK was female and 53% male [16]. Just under half of the respondents (48%, *n* = 143) were based in district general hospitals, 36% (*n* = 106) were working in university teaching hospitals, 4% (*n* = 11) worked exclusively in the private sector and 12% (*n* = 36) did not indicate the sector in which they worked. Since doctors enter into the specialty at varying ages, we did not record our respondents’ age, but rather how many years they have been at the consultant grade. We found that male respondents had practiced for more years than their female colleagues. While 60% of female consultants had less than 10 years experience, 72% of males had 10 years or more experience. This is also in line with the experience status of the current consultant cohort [16].

Thirty-nine percent (*n* = 117) described themselves as generalists in Obstetrics and Gynecology, and 15% (*n* = 44) responded that they were sub-specialists in Minimal Access Surgery (MAS) (Table 1.).

**Table 1:** How the respondents defined themselves

	%	Total number of responses (*N*)
Generalist	39	117
Minimal access surgeon	15	44
Urogynecologist	8	25
Reproductive medicine specialist	8	25
Gynecological oncologist	5	15
Non subspecialist obstetrician	2	7
Non subspecialist gynecologist	6	19
Missing response	12	36
Total	100	299

### Performance of myomectomy for symptomatic fibroids

With regard to performing myomectomy, 81% (*n* = 243) stated that they performed some type of myomectomy. Open myomectomy was the procedure performed by the vast majority (74%, *n* = 221). Hysteroscopic procedures were performed by 56% (*n* = 166), and 32% of the respondents (*n* = 95) stated that they perform laparoscopic myomectomy, while 29% (*n* = 86) of our respondents reported that they perform all three types of myomectomy.

Three fifths (46%, *n* = 138) of respondents reported that the size of the uterus would not influence their decision whether or not to perform open myomectomy. There is no limit on the number of fibroids removed laparoscopically by 18% (*n* = 54) and hysteroscopically by 31% (*n* = 93). Only 43% (*n* = 127) aim to remove all fibroids during an open myomectomy.

Male consultants are significantly more likely to perform a myomectomy compared to their female counterparts (90%, *n* = 137 males versus 69%, *n* = 74 females). Logistic regression modeling confirmed that this association was significant. Binary logistic regression modeling also showed that the experience and subspecialty/special interests of the consultants had a significant impact on their choice of performing or not performing myomectomy (Table 2).

**Table 2:** Key drivers for not performing myomectomy

Variables	SE	Sig.	OR
Female (compared to male)	*.459*	*.001*	*.232*
Experience 0–9 years (compared to 10 years+)	*.464*	*.012*	*.312*
Obstetrics and Gynecology (compared to Generalist)		.001	
Obstetrics (non-subspecialist) only	14,129.622	.999	.000
Gynecology (non-subspecialist) only	.672	.078	.306
Subspecialist/special interest in Gynecological Oncology	.861	.496	.556
Subspecialist/special interest in Urogynecology	*.645*	*.003*	*.146*
Subspecialist/special interest in Reproductive Medicine	.835	.975	1.027
Subspecialist/special interest in Fetal Maternal Medicine	*.842*	*.000*	*.035*
Subspecialist/special interest in Minimal Access Surgery	1.066	.108	5.554
Constant	.533	.000	32.073

Coefficients significantly different from zero at the 0.05 level or better are shown in italics

*SE* Standard error, *Sig* Significance, *OR* odds ratio

Consultants with more experience are significantly more likely to perform myomectomy than their colleagues with less than 10 years’ experience at consultant level (OR = 0.312, *p* = 0.012). Similarly, male consultants are also significantly more likely to operate compared to their female counterparts (OR = 0.232, *p* = 0.01). Perhaps unsurprisingly, respondents who described themselves as fetal medicine specialists operate significantly less than those in other subspecialties. The majority of respondents (89%, *n* = 194) agreed that complex fibroid surgery should only be performed by experienced gynecologists who frequently perform this operation.

### Attitudes towards myomectomy and women’s wishes

Fifty-four percent (*n* = 160) of our respondents said that they would offer a myomectomy to a woman whose family is complete, but who simply wishes to retain her uterus because she feels a more “complete woman”. Similarly, 175 respondents (59%) stated that they would perform a repeat myomectomy on a woman who wished to retain her fertility.

### Preventing and minimizing blood loss, the use of GnRH analogues (GnRHa), uterine artery embolization (UAE), and ulipristal acetate (UPA)

Although used in women undergoing all three types of myomectomy, the responses to our survey show that 86% (*n* = 257) of consultants use GnRHa prior to open myomectomy. Twenty-one percent (*n* = 62) used GnRHa prior to laparoscopic myomectomy while 44% (*n* = 131) use GnRHa prior to hysteroscopic myomectomy. The indications stated for GnRHa use were to correct anemia prior to surgery (50%, *n* = 148) and to reduce fibroid size to allow a lower transverse incision rather than a midline one (51%, *n* = 151). Interestingly, 41% (*n* = 122) stated that they use them despite knowing that GnRHa destroy tissue planes, thus rendering surgery more difficult.

Thirty percent (*n* = 91) of our respondents reported that they cross match blood for every open myomectomy they perform. Vasopressin was used by 29% (*n* = 86), while tourniquets/clamps were used by 20% (*n* = 57) to minimize blood loss during open myomectomy. Fifteen percent reported that they do not use any specific technique, and 44% (*n* = 132) have access to a cell salvage facility (Table 3).

**Table 3:** Methods used to reduce intraoperative blood loss

Methods used to minimize blood loss	*N*	%
Vasopressin	86	29
Tourniquets/clamps	57	19
Tranexamic acid	37	12
Oxytocin	3	1.0
Misoprostol	1	0.3
No method used	43	14
Missing	72	24
Total	299	100

Sixty-two percent (*n* = 184) of the respondents have access to UAE for the treatment of symptomatic uterine fibroids. When asked whether they would offer UAE to women wishing to conceive, 43% (*n* = 127) replied that they would. Forty-nine percent (*n* = 146) have read the joint guidelines published by The Royal College of Obstetrics and Gynecologists/Royal College of Radiologists [17] on the use of UAE for the treatment of uterine fibroids.

Seventy-three percent (*n* = 218) reported that they were aware of UPA, and 32% (*n* = 96) had used it pre-myomectomy. On the other hand, 29% (*n* = 87) respondents had never used UPA, while a mere 10% (*n* = 29) had used UPA for treating conditions other than symptomatic uterine fibroids, such as emergency contraception.

## Discussion

With a response rate of 22%, we are acutely aware of the need to interpret our findings with caution. However, the correlation between the results obtained in our survey with published gender breakdown of current consultants suggests that our respondents reflect the make up of consultants in the UK [16], and therefore, our findings provide a useful snapshot of current views and practices in the management of fibroids.

Although surveys are an important tool in the evaluation of health services, it would appear that response rates to most surveys have been declining over the years. Historically, surveys were conducted via the post, with apparently good responses. Thus, a postal survey on the same issue of myomectomy conducted by Taylor et al. [15] 10 years ago yielded a response rate of 54%. One would have assumed that the advent of the internet, with access to online surveys and the advantages of instant survey delivery, real-time data collection, ease of access for respondents, and increased respondent anonymity would increase the response rates for surveys. This does not appear to be the case, and it is reported that response rates differ among various specialties [18]. The ease of use of e-surveys may in fact be a factor contributing to the decline in response rates, as people are bombarded with a multitude of surveys, and it is so easy to delete the e-mail without even opening it. In a meta-analysis of 68 web-based health surveys, the mean response rate reported in 49 studies was 39.6% [19]. However, our own recent experience of a 28% response rate from a postal survey [20] would suggest that there is indeed a general decline in response rates, and this is mirrored in the experience of other colleagues (Sultan et al., personal communication). Thus, many factors probably contribute to the declining response rates, and it is unlikely that this trend can be improved significantly.

Despite the emergence of minimally invasive techniques, including UAE, and the pharmacological agent UPA, our work suggests that surgery is still the most common form of fibroid treatment offered in the UK, a finding supported by work published by the Health and Social Care Information Centre, Hospital Episode Statistics (HES) [21].

From their postal survey published 10 years ago, Taylor et al. [15] reported that 64% of their respondents performed at least one open myomectomy per year; with around half (51%) performing between one and five procedures annually. Our own survey suggests that the number of surgeons reporting that they performed between one and ten myomectomies per year has increased to 62%. Similarly, the rate of laparoscopic myomectomy has risen from 11 to 32%. Whether this is a reflection of the increased demand for uterine sparing procedures or has resulted from surgeons having a higher caseload or both remains to be answered.

The use of pre-myomectomy GnRHa use has not changed in the intervening 10 years: Taylor et al. [15] reported that 85% of their respondents use GnRHa pre-myomectomy, and our own survey yielded a similar result of 86%. Interestingly, 41% of our respondents stated that GnRHa destroy tissue planes and thus render the surgery more difficult. The Cochrane review on the preoperative use of GnRH comments that “fibroid capsule will become less evident and may be missed, tumours will not “shell out” cleanly and the excision may be more difficult” [22]. The literature also reports that the use of GnRHa is not cost-effective [23], delays surgery, and is associated with significant side effects and bone demineralization, and there are much cheaper alternatives such as progestogens to prevent menstruation while correcting anemia [24]. Further evidence is emerging regarding the preoperative use of UPA which could be a better alternative to GnRH: a recent study from Canada investigated the surgical experience of patients that underwent laparoscopic or robotic myomectomy following UPA treatment and found no difference compared to non-treated patients [25]. On a similar note, Sancho et al. described a case series where no difficulty was observed during hysteroscopic fibroid resection following UPA treatment [26].

Only one in five of the respondents to our survey reported that they used tourniquets or clamps. This is despite reports that the application of tourniquets is more effective than GnRHa in minimizing blood loss [27]. Our survey found that perioperatively, 29% (*n* = 86) of gynecologists use vasopressin to minimize blood loss. This relatively low rate may reflect concerns that have been raised recently concerning the safety of vasopressin [28].

Taylor et al. [15] reported that only 28% of their respondents were prepared to perform a myomectomy on a 50-year-old woman, presumably on the assumption that they no longer needed their uterus. While we did not ask this exact same question, 54% of respondents to our survey replied that they would perform a myomectomy on a woman who has completed her family, and 59% would perform a repeat myomectomy. These responses may reflect an increasing awareness and acceptance of women’s autonomy, as well as being driven by the changing demography of many women delaying childbirth until later in life.

Transformed attitudes may also be reflected in the increased awareness and acceptance of UAE seen in our survey. Taylor et al. [15] reported that only around half of their respondents had ready access to UAE. Our work suggests that this figure has risen to 62%. In the past, it was a common opinion that UAE should not be offered to women wishing to conceive, and this is reflected in 26% of the participants in Taylor et al.’s study who agreed that they would offer UAE to such women. With ever increasing reports of women having successful pregnancies following UAE, guidance is no longer quite so didactic, and in our survey, 43% of all our respondents would offer UAE to women planning conception. It is interesting to note that the advent of UAE and other uterus-sparing treatments have not reduced the number of myomectomies being carried out, perhaps another reflection that women have been demanding more uterine saving procedures compared to a decade ago.

According to the Centre for Workforce Intelligence’s latest report [16], the number of women in the Obstetrics and Gynecology workforce has increased more than fourfold, from around 200 in 1998 to over 893 in 2013. In the last few years, over four fifths (81%) of specialist trainees in Obstetrics and Gynecology are female. In our survey, three fifths (59%) of respondents were male. Of the male respondents, 90% reported that they performed some type of myomectomy, while the figure is significantly lower among females. The percentage of male consultants performing myomectomy with 10 years or more experience is 90% whereas the corresponding figure for females is 74% (*p* = 0.003). Among those with less than 10 years experience, the contrast is even starker as only 64% of females perform myomectomy compared to 91% of male consultants (*p* = 0.016). This raises an important question: is the lower surgery rate seen in women in our survey a result of their relative inexperience, or does this signify a paradigm shift away from surgery that female gynecologists deem unnecessary and towards the use of other primary treatments for symptomatic uterine fibroids? If the former, then our survey raises an important issue, which has major implications for training and workforce planning, namely, who will perform the myomectomies of tomorrow?

If we are to offer our patients the best outcomes, then any treatment of significant complexity should be delivered by a highly trained individual who administers that treatment on a frequent basis. Our specialty has seen a move away from “generalists” and towards dedicated sub-specialists such as those treating gynecological cancers, women with urinary disorders, and high-risk pregnancies. Fibroid surgery can be challenging, and it is informative that 89% of all our respondents agreed that only experienced gynecologists who perform these operations frequently should undertake complex fibroid surgery. Increasing rates of both open and minimally access surgery rates support our views that there is a demand for a subspecialist competent in all forms of myomectomy. As fibroids present in a wide range of sizes and locations with variety of symptoms, and patients may wish to have a selection of treatments; we believe that there simply is no single ideal treatment. Case-by-case assessment of each individual patient is therefore necessary to optimize the treatment where the role of experienced benign gynecological surgeon is indisputable.

## Conclusions

The changing demography of childbirth, with women increasingly postponing pregnancy to an age when fibroids are more numerous and symptomatic, poses a variety of challenges. There is a need to research and optimize the treatment options available, a cadre of highly skilled surgeons will be required, and this has major implications for the training of gynecologists, and new treatments must be sought that enhance fertility potential and symptom relief with minimal risk to the woman. Surveys such as the one presented here provide crucial snapshots of current practices and views and hopefully help to shape those practices and future directions.

## Additional file

Additional file 1: Copy of treating symptomatic uterine fibroids with myomectomy. (PDF 255 kb)

## Abbreviations

GnRHa: Gonadotrophin-releasing hormone analogue
UAE: Uterine artery embolization
UPA: Ulipristal acetate

## Footnotes

1 There were 2225 consultants employed in 154 NHS units as of 2013. https://www.rcog.org.uk/globalassets/documents/careers-and-training/census-workforce-planning/census-report-2013.pdf.
