# Supplementary material for: Treating symptomatic uterine fibroids with myomectomy: current practice and views of UK consultants
Source: Gynecol Surg. 2017 Jul 6;14(1):11. doi: 10.1186/s10397-017-1014-4 (PMC5570799; doi:10.1186/s10397-017-1014-4)
Supplement: Additional file 1: — Copy of treating symptomatic uterine fibroids with myomectomy. (PDF 255 kb) [file 10397_2017_1014_MOESM1_ESM.pdf]

Copy of Treating symptomatic uterine fibroids with myomectomy

1. Treating symptomatic uterine fibroids with myomectomy: Current views and...

Dear Colleague
We would like to learn your views and practices on treating symptomatic uterine fibroids with myomectomy. We are aware that you receive many requests to complete questionnaires, and apologize for this imposition. We estimate that this questionnaire will take 10 minutes of your valuable time, and we thank you in anticipation.

2.

1. Approximately how many myomectomy procedures do you perform per year?

	0	1-10	11-20	21-50	>50
Open /abdominal	○	○	○	○	○
Laparoscopic	○	○	○	○	○
Hysteroscopic	○	○	○	○	○

3.

2. Do you use pre-myomectomy GnRH analogues ?

	n/a	never	sometimes	All the time
Open/ abdominal	○	○	○	○
Laparoscopic	○	○	○	○
Hysteroscopic	○	○	○	○

3. How long do you use GnRH analogues for?

	n/a	1 month	2 months	3 or more months
open/abdominal	○	○	○	○
laparoscopic	○	○	○	○
hysteroscopy	○	○	○	○

4. Please look at the following statements as to reflect your views on the use of GnRH analogues prior to abdominal myomectomy

	Yes	No
I use it to correct anaemia prior to surgery	○	○
I use it reduce blood loss during surgery	○	○
It renders the operation easier	○	○
It destroys tissue planes	○	○
I use it reduce fibroid size to perform a transverse supra-pubic incision instead of a vertical incision	○	○
I use it to reduce fibroid size so to perform a vaginal hysterectomy instead of an abdominal one	○	○

4.

5. Do you cross-match blood prior to myomectomy ?

	n/a	Never	Sometimes	All the time
Open /abdominal	○	○	○	○
Laparoscopic	○	○	○	○
Hysteroscopic	○	○	○	○

6. Which of the following do you use at myomectomy to reduce blood loss?Please select all that applies

☐ Tourniquets/ clamps

☐ Vasopressin

☐ Misoprostol

☐ Oxytocin

☐ Tranexamic acid

☐ Other (please specify)

7. Do you use cell salvage at open abdominal myomectomy

○ Yes

○ No

5.

8. Is there is a limit on the size of uterus on which YOU would perform an abdominal myomectomy?

- n/a
- no limit
- up to 16/40weeks
- up to 20/40 weeks
- up to 24/40 weeks

9. Do you have a limit on the number of fibroids YOU would attempt to remove laparoscopically?

- n/a
- never more than 1
- maximum 2
- maximum 3
- maximum 4
- no limit

10. Do you have a limit on the size of intramural fibroids YOU would attempt to remove laparoscopically?

- n/a
- up to 5cm
- up to 10cm
- up to 20cm
- no limit

11. Do you have a limit on the number of fibroids YOU would attempt to remove hysteroscopically?

- n/a
- no more than 1
- maximum 2
- maximum 3
- maximum 4
- no limit

12. Do you have a limit on the size of fibroids YOU would attempt to remove hysteroscopically?

- n/a
- up to 2cm
- up to 4cm
- up to 6cm > 6cm

13. With regards to removal of fibroids at open myomectomy, how many fibroids do you aim to remove?

- n/a
- all of them
- only those that I believe are causing symptoms

6.

14. Would you perform a myomectomy on a woman whose family is complete, but who simply wishes to retain her uterus because she feels more a "complete" woman?

- Yes
- No

15. In a woman who had a previous open myomectomy who wishes to retain fertility potential, would you agree to perform a repeat myomectomy?

- Yes
- No

16. Should complex myoma surgery be performed only by experienced gynaecologists who frequently perform this operation?

- Yes
- No

7.

17. Do you have local access to a Uterine Artery Embolization (UAE) service?

- Yes
- No

18. Would you offer UAE to a woman wishing to conceive?

- Yes
- No

19. Have you read the latest (December 2013) RCOG /Royal Collage of Radiologists guideline on UAE?

- Yes
- No

8.

20. Please look at the following statements regarding your views / practices with the new drug Ulipristal Aceetate (Esmya)

	N/A	Yes	No
I have never heard of this drug	○	○	○
I have heard of this drug but I have never used it	○	○	○
I have used this drug on a number of occasions prior to myomectomy	○	○	○
I have used this drug pre-myomectomy and found it effective / useful	○	○	○
I now routinely use this drug pre-myomectomy	○	○	○
I have used ulipristal acetate as an emergency contraception	○	○	○
I have used ulipristal acetate for indications other than fibroids and emergency contraception	○	○	○

9. about yourself

21. How many years have you been a consultant?

○ 0 – 4

○ 5 – 9

○ 10 – 14

○ 15 - 19

○ >20

22. Please state your gender

○ Female

○ Male

23. Which of the following best describes your current place of practice?

- Tertiary Centre
- University Teaching Hospital
- District General Hospital
- Community / Primary Care setting
- I am retired

24. Which of these options best describes your current field of work?

- Obstetrics (non-subspecialist) only
- Gynaecology (non-subspecialist) only
- Obstetrics & Gynaecology (generalist)
- Subspecialist in Gynaecological Oncology
- Subspecialist in Urogynaecology
- Subspecialist in Reproductive Medicine
- Subspecialist in Fetal Maternal Medicine
- Other (please specify)

10. Thank you

Thank you for your time. If you have any further comments please e-mail fsirkeci@sgul.ac.uk.
